# Effects of Hfq on the conformation and compaction of DNA

**DOI:** 10.1093/nar/gkv268

**Published:** 2015-03-30

**Authors:** Kai Jiang, Ce Zhang, Durgarao Guttula, Fan Liu, Jeroen A. van Kan, Christophe Lavelle, Krzysztof Kubiak, Antoine Malabirade, Alain Lapp, Véronique Arluison, Johan R.C. van der Maarel

**Affiliations:** 1 Department of Physics, National University of Singapore, 2 Science Drive 3, 117542, Singapore; 2 Genomes Structure and Instability, Sorbonne Universities, National Museum of Natural History, Inserm U 1154, CNRS UMR 7196, 75005 Paris, France; 3 Laboratoire Léon Brillouin, UMR 12 CEA/CNRS, CEA-Saclay, Gif sur Yvette Cedex 91191, France; 4 Department of Molecular Biology, University of Gdansk, Wita Stwosza 59, 80-308 Gdansk, Poland; 5 Université Paris Diderot, Sorbonne Paris Cité, 75013 Paris, France

## Abstract

Hfq is a bacterial pleiotropic regulator that mediates several aspects of nucleic acids metabolism. The protein notably influences translation and turnover of cellular RNAs. Although most previous contributions concentrated on Hfq's interaction with RNA, its association to DNA has also been observed *in vitro* and *in vivo*. Here, we focus on DNA-compacting properties of Hfq. Various experimental technologies, including fluorescence microscopy imaging of single DNA molecules confined inside nanofluidic channels, atomic force microscopy and small angle neutron scattering have been used to follow the assembly of Hfq on DNA. Our results show that Hfq forms a nucleoprotein complex, changes the mechanical properties of the double helix and compacts DNA into a condensed form. We propose a compaction mechanism based on protein-mediated bridging of DNA segments. The propensity for bridging is presumably related to multi-arm functionality of the Hfq hexamer, resulting from binding of the C-terminal domains to the duplex. Results are discussed in regard to previous results obtained for H-NS, with important implications for protein binding related gene regulation.

## INTRODUCTION

Hfq is a widespread and phylogenetically conserved protein found at high concentration in bacteria. Consistent with this high level of expression, the protein is central to multiple regulatory processes. For instance, the initial description of Hfq was associated with its involvement in the activity of bacteriophage Qβ replicase [hence its iconic name standing for host factor for phage Qβ RNA replication (1)]. However, pleiotropic functions of Hfq have been highlighted when the *hfq* gene was disrupted in *Escherichia coli*, yielding a decrease in growth rate, an increase in UV, oxidant and osmo sensitivity, as well as decreased negative supercoiling of plasmids (2). Most of these *hfq*-null phenotypes are due to the implication of Hfq in RNA regulation by small non-coding RNA (sRNA) (3). Indeed, Hfq is required to mediate sRNA stress-response. This regulatory mechanism is based on the hybridization of sRNA to its target mRNA, therefore altering mRNA expression. Most often, the hybridization of sRNA occurs in the vicinity of the ribosome binding site to prevent the initiation of translation, a process concomitant with a decrease in mRNA stability (4,5).

Similar to its eukaryotic homologs participating in pre-mRNA splicing and decay (6), Hfq is particularly important in RNA metabolism. It is structurally related to the Sm family of proteins. The amino-terminal region of Hfq forms a fold, which is also found in archaeal and eukaryotic Sm proteins (7). This N-terminal region (NTR) comprises five β-strands that form a bent β-sheet, capped by an α-helix. The β-sheets from six monomers interact with each other to assemble into a toroidal structure. The main difference between Hfq and Sm proteins is the number of polypeptides forming the core of the torus: whereas Sm proteins consist in heptameric rings, Hfq is composed of six polypeptides and is hexameric (8). Although the mechanism by which Hfq binds RNA is not completely understood, it is now well-established that the NTR of the protein (∼65 amino acids) binds RNA in the absence of the C-terminal region (CTR) (9), uridine-rich RNA sequences are bound to the proximal face, whereas purine-rich RNA sequences bind to the distal face of the torus (10,11). Furthermore, the hexamer exhibits a rim that has been proposed to participate in RNA binding (12).

As previously mentioned, in addition to its NTR domain forming the hexameric torus, the protein also includes a CTR domain of ∼35 amino acids. This domain appears mainly non-structured (13) and its function has not yet been resolved. Indeed, the importance of the CTR tail of Hfq is a matter of controversy. On one hand, some studies have shown that tail-less Hfq is efficient for RNA binding and regulation (9,14). On the other hand, other studies have suggested that a lack of the tail reduces RNA-mediated regulation (15–18) and that some residues in the CTR interact with RNA (19). Moreover, we previously observed that Hfq is able to self-assemble and that this ability is dependent on the presence of the CTR (20). This is in accordance with the recent observation that the CTR of Hfq is flexible, a feature which could facilitate protein–protein interactions (13).

In addition to its role in post-transcriptional regulation, Hfq is capable of binding to DNA, but with a weaker affinity than to RNA (21–24). This was notably emphasized in cellular localization experiments, demonstrating that ∼20% of Hfq is complexed with DNA (25,26), along with a tenth of other DNA-binding proteins within the bacterial nucleoid (23,27–28). Hfq appears to have a heterogeneous localization in the nucleoid, as opposed to the more uniform distribution of most nucleoid associated proteins such as H-NS (histone-like nucleoid structuring protein) or HU (heat-unstable nucleoid protein) (25). A few studies have shed light on the importance of Hfq in DNA metabolism, as constraining negative supercoiling *in vivo* (2) or signifying its role in transcription (29–31), replication (32) and transposition (33,34). Motivated by this unresolved role of Hfq, here, we investigate the effect of Hfq on the conformation and compaction of DNA using nanofluidics and atomic force microscopy. In parallel, information on the structural arrangement of bound Hfq on DNA is obtained with small angle neutron scattering (SANS).

A relatively new technology for the investigation of single DNA molecules and their complexes with ligands and proteins is nanofluidics. Quasi one-dimensional channels with cross–sectional diameters of one to a few hundred nanometer in combination with fluorescence microscopy can be used to study the conformation and compaction of single DNA molecules (35–37). In particular, it was shown that DNA confined inside such a nanochannel can be compacted into a condensed form for over-threshold concentrations of a neutral crowding agent (dextran) and like-charged proteins (bovine serum albumin and haemoglobin) (38–40). DNA condensation with DNA-binding proteins protamine and H-NS has also been reported (41,42). Here, bacteriophage T4-DNA molecules are pre-incubated with Hfq and brought into an array of nanochannels of various cross-sectional diameter. Information on the bending rigidity (persistence length) of the nucleoprotein complex and Hfq mediated bridging between different segments of the DNA molecule is then derived from the measurement of the stretch of well-equilibrated DNA molecules along the direction of channels, as well as the determination of the critical concentration of Hfq for the compaction of DNA into the condensed form. For reference, we also measure the size and compaction of unconstrained DNA molecules in the bulk phase.

The results obtained with nanofluidics regarding the bending rigidity and Hfq mediated bridging are corroborated with atomic force microscopy. Atomic force microscopy is particularly useful to study protein–DNA interaction, because the complexes are directly visualized without relying on elaborate sample preparation. Here, we investigate the effect of Hfq on the persistence length from an analysis of the orientation correlation of single DNA molecules adsorbed on a silica surface without additional components to promote binding (43). Furthermore, we trace the length of the molecules along the contour to gauge a possible effect of Hfq on the rise per bp along the helical axis. In order to avoid complications related to looping, bridging and side-by-side aggregation, we use relatively short DNA fragments of 1000 bps. Hfq mediated bridging is confirmed, however, by imaging of longer DNA fragments consisting of 10 000 bps.

Nanofluidics and atomic force microscopy are not well adapted to the investigation of the structural arrangement of a protein about DNA. Typical structure-related properties are best and quantitatively inferred from scattering experiments. In the case of DNA, scattering studies have focused on the radial density profile of counterions away from the DNA axis (44–48). To the best of our knowledge, ordering of DNA-binding protein has never been investigated before by similar methods. An advantage of SANS is the possibility for variation of DNA and protein contrasts (49). In this contribution, we apply contrast variation in mixtures of H_2_O and D_2_O to obtain the scattering from the protein in solutions of persistence length (50 nm) DNA fragments. The scattering is analyzed in terms of a radial distribution of Hfq around DNA, to arrive at a consistent model of the binding of the Hfq hexamer on DNA and the formation of bridges with CTR multi-arm functionality.

Our results enable to propose a mechanism underlying the function of Hfq in coordinating DNA packaging and unveil a new role for this pleiotropic factor in the control of gene expression.

## MATERIALS AND METHODS

### Sample preparation

T4 GT7 DNA (T4-DNA, 165.65 kbp) was purchased from Nippon Gene, Tokyo and used without further purification. The integrity of T4-DNA was verified with pulsed gel electrophoresis. No fragments of ones to tens of kbps were observed. Hfq (*M*$w$ = 67 kDa, hexamer) was purified from BL21(DE3)/pTE608 cells expressing untagged Hfq as previously described (50). Samples were prepared by dialyzing solutions of DNA against 10 mM Tris–HCl (T) buffer with the relevant concentration of NaCl and/or MgCl_2_ in micro dialyzers. For one sample series, NaCl was substituted by potassium glutamate (KGlu). Solutions of Hfq in the same buffer were also prepared. The Tris–HCl concentration is 10 mM Tris adjusted with HCl to *p*H 7.5 (i.e. 8.1 mM Tris–Cl and 1.9 mM Tris). The ionic strength of the buffer was calculated with the Davies equation for estimating the activity coefficients of the ions and a dissociation constant *p*K = 8.08 for Tris. Solutions of Hfq and DNA were subsequently mixed and incubated for 12 h at 277 K. YOYO-1 fluorescence staining dye was purchased from Invitrogen, Carlsbad, CA, USA. DNA was stained with YOYO-1 with an incubation time of 12 h and an intercalation ratio of 100 bps per dye. No anti-photo bleaching agent was used.

### Electrophoretic mobility shift assay

The binding of Hfq to DNA was investigated with a gel shift assay. A 250-bp DNA fragment, including a 20 A-tract, was produced by polymerase chain reaction. The fragments (30 nM) were incubated with YOYO-1 (50 bps per dye) and various concentrations of Hfq (0.2–5 μM) and electrophoresed on a 1.5% agarose gel. A control experiment was done without YOYO-1. The gel was stained with SYBR green I and imaged with a G:BOX system (Syngene, Cambridge, UK).

### Chip fabrication

The nanofluidic devices were fabricated by replication in polydimethylsiloxane (PDMS) of patterned master stamps (51,52). The nanochannels were made in hydrogen silsesquioxane (HSQ) resist (Dow Corning, Midland, MI, USA) using a lithography process with proton beam writing (53). An array of nanochannels is connected to two loading reservoirs through a superposing set of microchannels made in SU-8 resist with UV lithography. The heights and widths of the ridges in the master stamps were measured with atomic force microscopy (Dimension 3000, Veeco, Woodbury, NY) and scanning electron microscopy, respectively. Two stamps were made featuring nanochannels of length 60 μm and rectangular cross-sections of 150 × 250 and 200 × 300 nm^2^, respectively. The stamp was coated with a 5-nm thick teflon layer to guarantee perfect release of the replicated PDMS chips (54). The stamps were replicated in PDMS followed by curing with a curing agent (Sylgard, Dow Corning) at 338 K for 24 h. The PDMS replica was sealed with a glass coverslip, after both substrates were plasma oxidized (Harrick, Ossining, NY).

### Nanofluidics

The pre-incubated and stained DNA molecules were loaded into one of the two reservoirs connected to the array of nanochannels. The DNA molecules were subsequently driven into the channels by electrophoresis. For this purpose, two platinum electrodes were immersed in the reservoirs and connected to a power supply with a relatively low voltage in the range 0.1–10 V (Keithley, Cleveland, OH, USA). Once the DNA molecules were localized inside the nanochannels, the electric field was switched off and the molecules were allowed to relax to their equilibrium state for at least 60 s. The stained DNA molecules were visualized with a Nikon Eclipse Ti inverted fluorescence microscope equipped with a 200 W metal halide lamp, a filter set, and a 100× oil immersion objective. A UV light shutter controlled the exposure time. Images were collected with an electron multiplying charge coupled device (EMCCD) camera (iXon X3, Andor Technology, Belfast, UK) and the extension of the DNA molecules inside the channels was measured with imageJ software (http://rsb.info.nih.gov/ij/). For intensity threshold, we have used two times the signal to background noise ratio.

### Bulk phase imaging

A droplet of solution was deposited on a microscope slide and sealed with a coverslip separated by a 0.12 mm spacer. The YOYO-1 stained T4-DNA molecules were imaged with the above mentioned microscope. One minute video clips were collected with a frame rate of five frames per second. The video clips were analyzed with matlab (Natick, MA, USA). For each frame (time *t*), the radius of gyration tensor of the imaged molecule was constructed according to **S**(*t*) = ∑_*m, n*_(**r**_*mn*_ − **r**_*cm*_)^2^*I*_*mn*_/*I*_0_, with *I*_*mn*_ the fluorescence intensity of pixel [*m, n*] at position **r**_*mn*_ in the *xy*–plane and *I*_0_ is the total intensity of the frame. The center of mass **r**_*cm*_ of the molecule was calculated according to **r**_*cm*_ = ∑_*m, n*_**r**_*mn*_*I*_*mn*_/*I*_0_. We determined the principal eigenvalues and vectors of **S** using a singular value decomposition and with the assumption of cylindrical symmetry. We subsequently derived the length of the long axis according to $L_{\Vert }= 12 \, < {S}_\Vert >$, where ${S}_\Vert$ represents the largest eigenvalue and the brackets denote an average over all frames.

### Atomic force microscopy

DNA fragments were purchased from Thermo Scientific (1000 bp, Waltham, MA, USA) and New England Biolabs (10 000 bp, Ipswich, MA, USA). All imaging experiments were done at room temperature in air with a Veeco Dimension 3000 atomic force microscope (Woodbury, NY). Images were acquired in the tapping mode with silicon (Si) cantilevers (spring constant of 20–100 N/m) and operated below their resonance frequency (typically 230–410 kHz). The images were flattened, and the contrast and brightness were adjusted for optimal viewing conditions. A 20-μl droplet was spotted onto a silica surface. After 10 min to allow for DNA adsorption onto the surface, the specimens were developed by flushing them with ultra pure water followed by drying in a stream of N_2_ gas.

### Small angle neutron scattering

DNA fragments (150 bp) were obtained by micrococcal digestion of calf thymus chromatin (55). A set of solutions was prepared by dissolving DNA and Hfq in mixtures of H_2_O and D_2_O to a concentration of 10 and 140 g/l, respectively. The solutions also contained 100 mM KCl. We applied contrast variation with four solvent compositions: 0, 40 (Hfq matched), 64 (DNA matched) and 100% D_2_O. Small angle neutron scattering was measured at ambient temperature with the PAXY diffractometer situated on the cold source of the Laboratoire Léon Brillouin. A wavelength of 0.6 nm with a 10% spread was selected. The scattering contributions pertaining to DNA and Hfq are then obtained by a simultaneous two-parameter fit to the data from the four solvent compositions (46,48).

## RESULTS

### Nanofluidics

**Figure 1.**
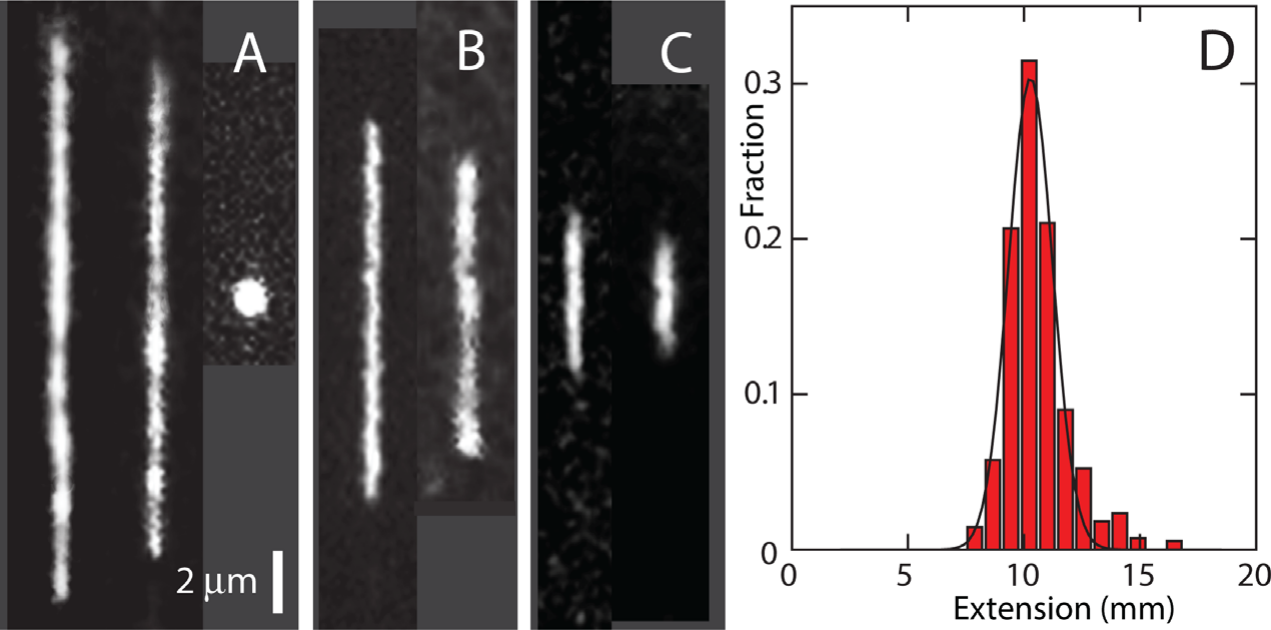
(**A**) Montage of fluorescence images of T4-DNA molecules inside 150 × 250 nm^2^ channels and in T-buffer. From left to right 2 × 10^−4^, 2 × 10^−3^ and 3.3 × 10^−1^ (condensed) μM Hfq. (**B**) T4-DNA inside 200 × 300 nm^2^ channels and in T-buffer with 2 × 10^−4^ (left) and 2 × 10^−3^ (right) μM Hfq. (**C**) T4-DNA inside 200 × 300 nm^2^ channels and in T-buffer with 30 mM NaCl. The Hfq concentrations are 2 × 10^−4^ (left) and 2 × 10^−3^ (right) μM. (**D**) Distribution in extension of a population of 30 molecules in T-buffer with 2 × 10^−3^μM Hfq. A Gaussian fit gives a mean extension *R*_∥_ = 10 ± 1 μm.

T4-DNA molecules with a concentration of 3 mg of DNA/l were incubated with the relevant buffer for at least 24 h. It was checked that the DNA molecules are in the coil state before they were brought into the channels of the nanofluidic device with an electric field. After the field is switched off, the molecules relax to their equilibrium state within 60 s. We have verified that there is no further change in stretch for more than 3 h. A montage of images of single DNA molecules confined inside rectangular channels is shown in Figure 1. The equilibrated stretch in the longitudinal direction of the channel decreases with increasing concentration of Hfq. For over-threshold concentrations of Hfq, condensation of DNA into a compact form is observed. Condensed DNA is visible as a bright fluorescence spot and can easily be discerned from the extended form. We observed the same qualitative behaviour, irrespective channel cross-section and ionic composition of the buffer. There are quantitative differences however in the values of the stretch, relative decrease with increasing concentration of Hfq and critical concentration of Hfq for condensation. For buffer molarities exceeding ∼40 mM (10 mM Tris/HCl with 30 mM NaCl or KGlu), fluorescence imaging of the DNA molecules becomes problematic because of quenching and reduced binding of the dye. Furthermore, at high ionic strength the stretch becomes too short for a reliable detection of the transition from the coil to the condensed state.

**Figure 2.**
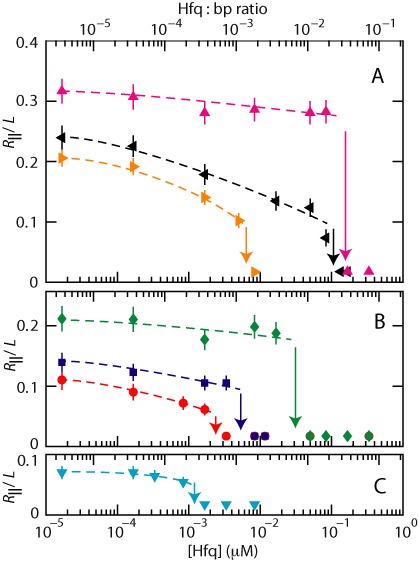
(**A**) Relative extension *R*_∥_/*L* of T4-DNA in T-buffer (magenta, Δ), T with 3 mM NaCl (black, ◃) and T with 30 mM KGlu (gold, ▹) versus the concentration of Hfq (bottom axis) and Hfq to bp ratio (top axis). The molecules are inside 150 × 250 nm^2^ channels. (**B**) As in panel (A), but for molecules inside 200 × 300 nm^2^ channels. The buffers are T (green, ⋄), T with 3 mM NaCl (blue, $\Box$) and T with 30 mM NaCl (red, ○). (**C**) As in panel (A), but inside 200 × 300 nm^2^ channels and in T-buffer with 3 mM NaCl and 0.4 mM MgCl_2_ (cyan, ▽). The dashed curves are drawn as an aid to the eye and the arrows denote the condensation thresholds.

We have measured the extension of the DNA molecules with two channel systems with channel cross-sections of 150 × 250 and 200 × 300 nm^2^, respectively. For each experimental condition, that is buffer composition, channel diameter and Hfq concentration, we have used a fresh PDMS replica and measured ∼30 molecules. The distribution in extension is close to Gaussian (56). An example of such a distribution is shown in Figure 1D. Fragmented DNAs can easily be discerned, because their extensions clearly fall below the values pertaining to the intact molecules. For the cut off, we have used the mean value minus two times the standard deviation. Resolution broadening can be neglected, because the optical resolution is one order of magnitude smaller than the variance. The mean relative extension *R*_||_/*L*, that is the mean extension divided by the contour length of 57 μm, is set out in Figure 2 as a function of Hfq concentration. With increasing concentration of Hfq, a relative decrease in extension by 10 to 60% is observed with the largest value for molecules bathed in a buffer of higher ionic strength. Due to the increased screening of electrostatic interaction, the extension decreases with increasing ionic strength. In the presence of MgCl_2_ or KGlu, the same qualitative behavior is observed. Note that for subthreshold concentrations of Hfq the relative extensions are in the range 0.05–0.3, which implies that the DNA molecules remain coiled. Furthermore, related to the stronger confinement, an increased stretch is observed in the 150 × 250 nm^2^ channel system.

For over-threshold concentrations of Hfq, the DNA molecules compact into a condensed form. This is facilitated by the confinement inside the nanochannel, because we did not observe condensation in the feeding microchannels and/or reservoirs of the chip. The critical concentration of Hfq for condensation decreases with increasing ionic strength and/or in the presence of a submillimolar concentration of MgCl_2_. Furthermore, the threshold shifts to a higher value with decreasing channel cross-sectional diameter. The highest observed critical concentration of Hfq for condensation inside 150 × 250 nm^2^ channels and at an ionic strength of ∼8 mM (T-buffer) corresponds with an Hfq to bp ratio of 1:40. The latter ratio decreases dramatically through hundreds to thousands of bps per Hfq hexamer for wider channels and with increased concentration of salts.

### Condensation in the bulk phase

**Figure 3.**
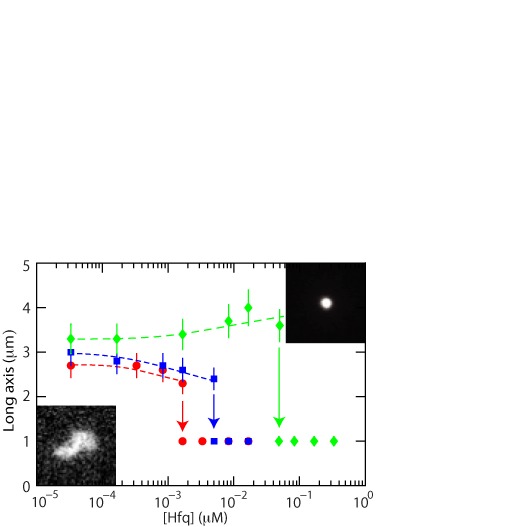
Long axis of unconstrained T4-DNA versus the concentration of Hfq. The buffers are T (green, ⋄), T with 3 mM NaCl (blue, $\Box$) and T with 30 mM NaCl (red, ○). The dashed curves are drawn as an aid to the eye and the arrows denote the condensation thresholds. The insets shows fluorescence images of a non-condensed (left) and condensed (right) T4-DNA molecule.

We have also investigated how the coil-size of unconstrained DNA molecules changes with the addition of Hfq. Fluorescence microscopy experiments were done with 0.03, 0.3 and 3 mg of T4-DNA/l in T-buffer and T-buffer with 3 and 30 mM of added NaCl (0.03 mg of DNA/l only). The molecules were observed to be slightly anisotropic. We measured the radius of gyration tensor and derived the length of the long axis of the molecules. The results, as well as some characteristic images, pertaining to 0.03 mg of DNA/l are shown in Figure 3 (notice that the nanofluidics experiments were done with a loading buffer containing 3 mg of DNA/l). Qualitatively, we observe the same behavior as for the measurement of the extension in nanochannels. A difference is that in the bulk phase and minimal screening conditions, the long axis slightly increases with increasing Hfq concentration. Furthermore, in the bulk phase we observed coexistent condensed and non-condensed molecules at the critical concentration of Hfq, in agreement with a discontinuous, first order transition. In the channel system, the transition appears to be continuous (38). Quantitatively, there are differences in the scale and critical concentration of Hfq for condensation. In the bulk phase, the critical concentration depends on the concentration of DNA. The critical concentrations are 0.05, 0.08 and 0.5 μM Hfq for 0.03, 0.3 and 3.0 mg of DNA/l, respectively (T-buffer). For the 200 × 300 and 150 × 250 nm^2^ channel systems, the corresponding critical concentrations are 0.03 and 0.1 μM Hfq, respectively (Figure 2). Condensation in the bulk phase requires, accordingly, an order of magnitude higher concentration of Hfq.

Fluorescence microscopy was done with a minimal level of staining of 100 bps per YOYO-1 molecule. For such a low staining level, there is no appreciable effect on the contour length, net charge and bending rigidity of the DNA molecules (51,57). To check whether there are complications associated with the staining, we have also done a series of experiments with 50 bps per dye. We obtained identical results for the stretch and condensation threshold. Furthermore, in an electrophoretic mobility shift assay (Supplementary Figure S1), the observed band shift is not affected by YOYO-1. Accordingly, staining of DNA with YOYO-1 in the range 50–100 bps per dye does not affect the formation of the Hfq–DNA complex.

### Contour and persistence length

**Figure 4.**
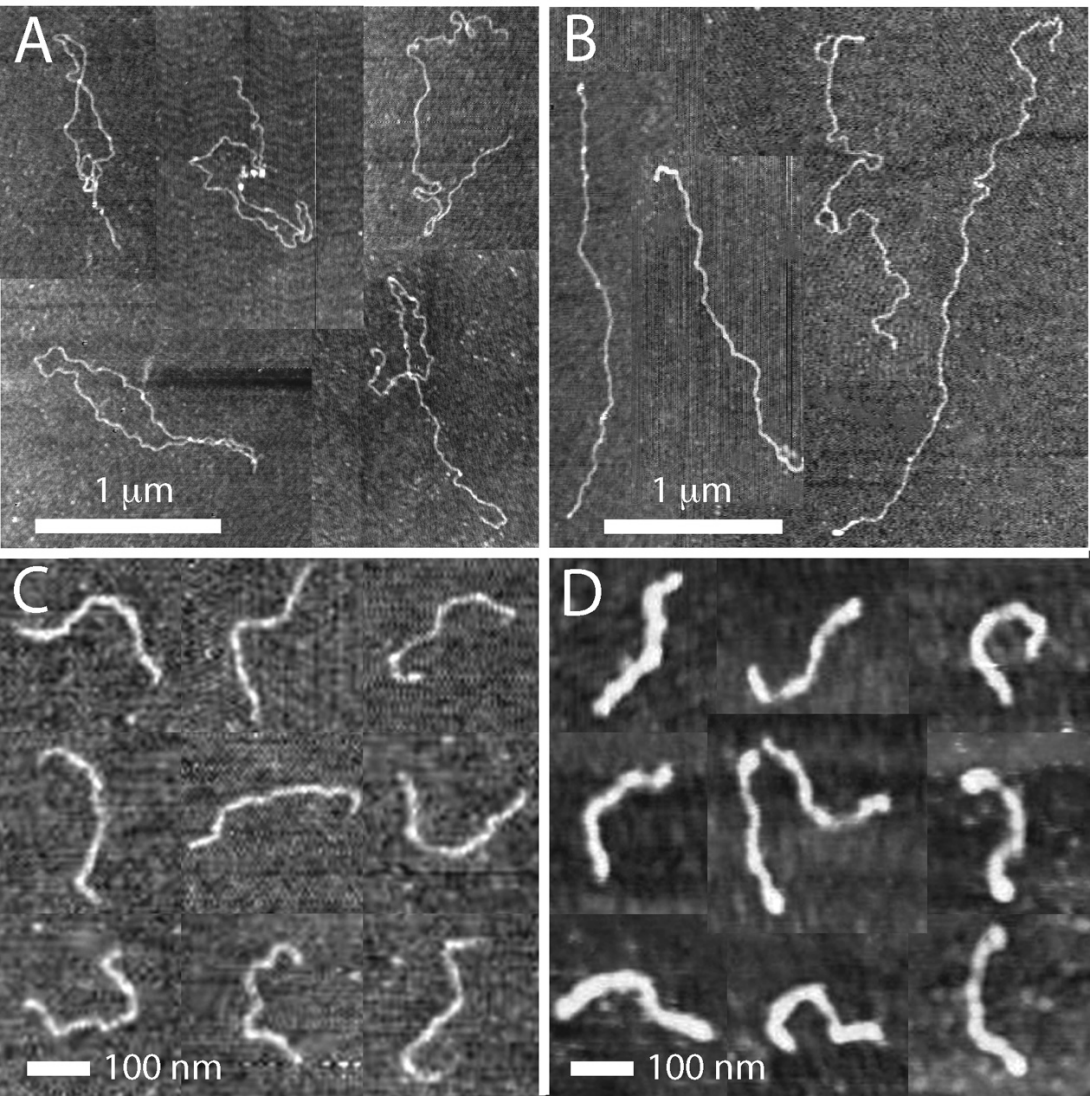
(**A**) Tapping mode atomic force microscopy images of DNA (10 000 bps) with Hfq to bp ratio of 1:300. (**B**) As in panel (A), but with Hfq to bp ratio of 1:15. (**C**) DNA (1000 bp) with Hfq to bp ratio of 1:300. (**D**) As in panel (C), but with Hfq to bp ratio of 1:6. Panels (A–D) are a montage.

Binding of Hfq might affect the conformation of DNA through a change in mechanical properties of the duplex. To check this out, we have investigated the contour and persistence length of Hfq-coated DNA with atomic force microscopy. Linear DNAs of 10 000 and 1000 bps were used at a concentration of 3 mg/l. The DNAs were incubated with 0.015, 0.30 or 0.75 μM Hfq for 12 h, which correspond with an Hfq to bp ratio of 1:300, 1:15 and 1:6, respectively. For each sample, a droplet of 5 μl was spotted onto a silica surface. The Hfq coated DNA molecules weakly adsorb to silica, so that no additions to the buffer are necessary to promote adhesion. Furthermore, the molecules equilibrate on the surface in a 2D conformation. Excess protein was removed by flushing the specimens with ultra pure water. Subsequently, the specimens were N_2_ dried. A series of typical images for increasing Hfq to bp ratio and for the two DNA molecular weights are shown in Figure 4.

For 10 000 bp DNA with an Hfq to bp ratio of 1:300 (Figure 4A), the length of the molecules measured along the contour agrees with the expected value based on molecular weight (3.4 μm). However, once the Hfq to bp ratio is increased to 1:15, intramolecular back folding, looping and side-by-side aggregation is observed (Figure 4B). The side-by-side aggregation is caused by Hfq mediated bridging of segments of the DNA molecule. Intramolecular aggregation complicates the analysis of the images for the determination of the contour and persistence lengths. As can be seen in panels C and D of Figure 4, shorter molecules of 1000 bps are visible as semi-flexible rods and do not exhibit aggregation nor looping. Accordingly, we have used the latter molecules to investigate the effect of binding of Hfq to DNA on the contour and persistence length of the nucleoprotein complex.

**Figure 5.**
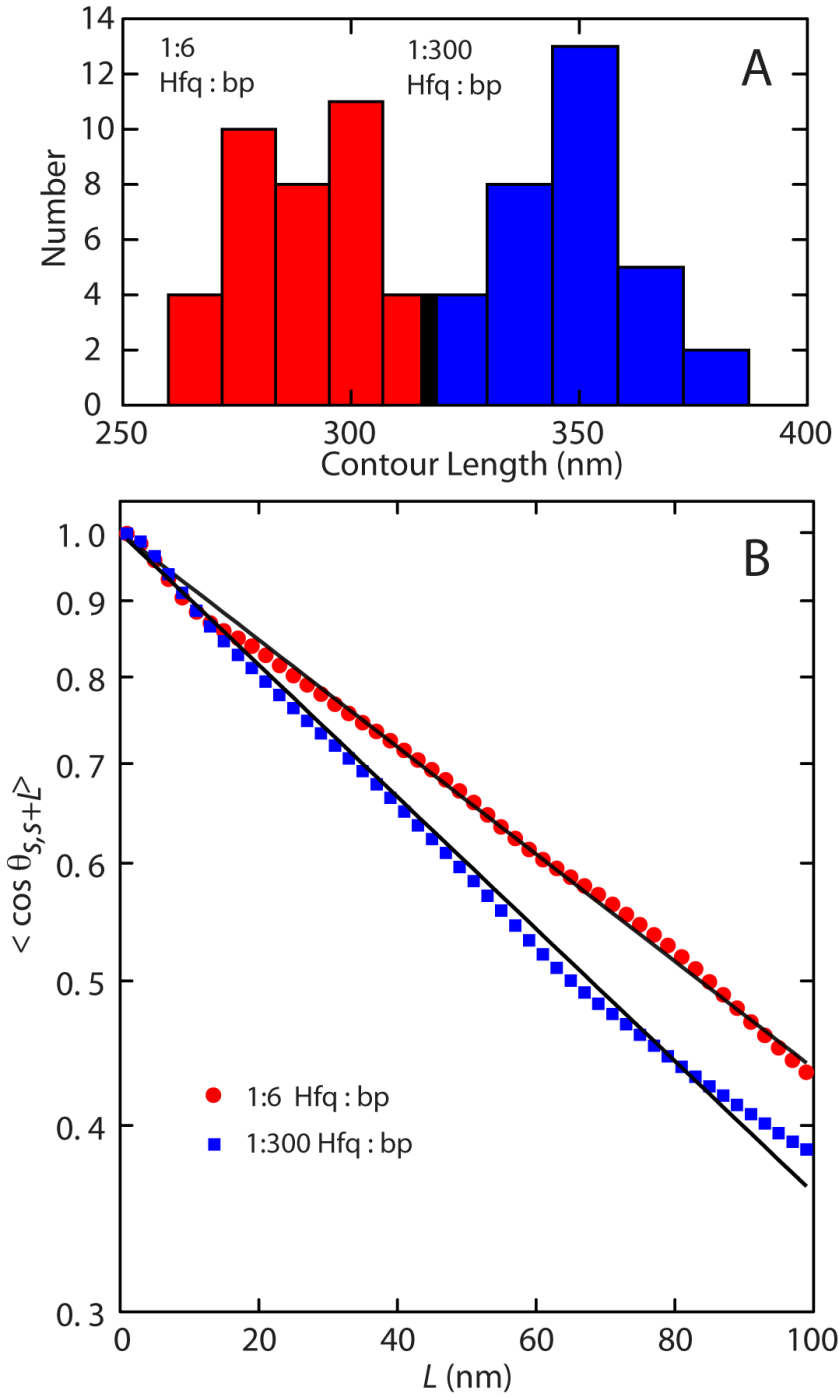
(**A**) Distribution in contour length of DNA (1000 bp) molecules with Hfq to bp ratio of 1:6 (red) and 1:300 (blue). (**B**) Orientation correlation function of the tangent vectors at a pair of points separated by distance *L* along the contour. The closed symbols are the experimental data obtained by averaging 31–37 molecules. The solid lines represent exponential fits.

To obtain the contour and persistence length, we traced the centerline of a population of 31–37 individual 1000 bp molecules. The distributions in length are displayed in Figure 5A. A significant reduction in contour length is observed if the Hfq to bp ratio is increased. In the case of one Hfq per 300 bps, the contour length agrees with the regular value of 350 ± 20 nm for double-stranded DNA in the B-form. However, with one Hfq per six bps, the contour length is decreased and takes a value of 270 ± 20 nm.

The centerlines were also used to obtain the tangent vector correlation function 〈cos θ_*s, s* + *L*_〉, where θ is the angle between tangent vectors at points *s* and *s* + *L*, by averaging *s* along the contour (43). For DNA molecules equilibrated in 2D conformation, this correlation follows:

(1) \begin{equation*} \langle \cos {\theta _{s,s+L}}\rangle = \exp \left[ {-L/(2P)} \right] \end{equation*}

The inverse of the exponential decay constant gives hence the persistence length *P*. Experimental and fitted tangent correlation functions are shown in Figure 5B. With increasing Hfq to bp ratio from 1:300 to 1:6, *P* increases from 49.1 ± 0.3 to 60.4 ± 0.3 nm. The value obtained for Hfq to bp ratio of 1:300 agrees with the commonly accepted value of 50 nm for bare DNA (43). The moderate increase in persistence length for coated DNA shows that the flexibility of the nucleoprotein complex is largely preserved. The images clearly show that the DNA molecules are coated with Hfq. The averaged heights of the complexes are 0.9 ± 0.1 and 2.6 ± 0.4 nm for Hfq to bp ratio of 1:300 and 1:6, respectively. These values are however indicative, because the complexes are dried and spread on the silica surface.

### Cross-sectional profile

**Figure 6.**
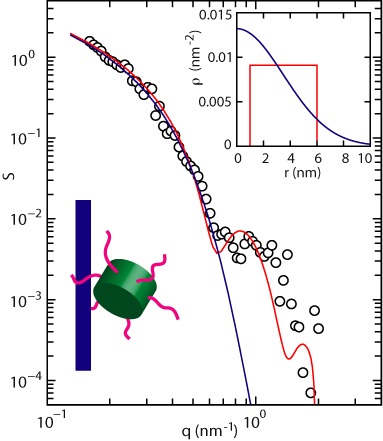
Protein structure factor *S* versus momentum transfer *q*. The blue and red curves represent the model calculations for a cylindrical complex with a Gaussian and shell-like radial distribution in amino acid density, respectively. The corresponding distributions are shown in the inset. The distribution in blue is Gaussian and provides a cross-sectional radius of gyration 5 nm. The distribution in red represents a shell-like protein coat with an inner and outer radius of 1 and 6 nm, respectively. The structure factor is normalized to *N*_*p*_ for *q* → 0. The cartoon illustrates a dangling Hfq hexamer bound on DNA (not to scale).

For more detailed structural information about the nucleoprotein complex, low resolution SANS experiments were done on a system of 150-bp DNA fragments and Hfq in 100 mM KCl (Hfq to bp ratio of 1:7.5). The contribution to the scattering from the protein (protein structure factor) is shown in Figure 6 as a function of momentum transfer *q*. Momentum transfer *q* is defined by the wavelength λ of the radiation and scattering angle θ between the incident and scattered beam according to *q* = 4π/λ  sin (θ/2).

The protein structure factor represents the spatial fourier transform of the amino acid density correlation function (49). Information on the structure of the nucleoprotein complex can be obtained by comparison of a coarse grained model calculation and the experimental, low resolution data. For this purpose, we consider the Hfq hexamer as the elementary scattering unit. Furthermore, we recognize that a fraction of Hfq is bound to DNA (*N*_*p*_ proteins per DNA molecule) and forms a cylindrical complex with a length *L* and radial distribution in amino acid density *ρ*(*r*). The distribution of Hfq along the DNA molecule is assumed to be homogeneous. Any possible ordering of Hfq in register with the phosphate moieties along the DNA molecule is beyond detection in scattering from solution with isotropic orientation averaging of the complex. For a cylindrical nucleoprotein complex, the protein structure factor takes the form

(2) \begin{equation*} S(q) = {\frac{\pi N_p}{qL}} a^2(q) \; \: \, \: (qL \gg 1) \end{equation*}

with *a*(*q*) the Hankel transformation of the radial amino acid density profile, that is

(3) \begin{equation*} a(q)= \int _{0}^{\infty } 2 \pi \, r\, J_0 (qr) \, \rho (r)\,dr \end{equation*}

and *J*_0_ denotes the zero order Bessel function of the first kind. Notice that the structure factor is normalized to *N*_*p*_/*L*, that is the inverse interspersion length of bound protein along the contour. Free protein does not significantly contribute to the scattering at small angles, because of lack of long range order.

For the amino acid distribution in the radial direction away from the spine-axis of the nucleoprotein complex, we have used two different models. In the first model, we assume a Gaussian profile $\rho (r) = \exp ({-r^2/ { r_p^2 }})/ ({\pi r_p^2 })$ with cross-sectional radius of gyration *r*_*p*_. The corresponding model calculation with optimized *r*_*p*_ = 5.0 ± 0.5 nm is shown in Figure 6 (the radial profile is shown in the inset). The use of a Gaussian profile results in a reasonable fit of the protein structure factor in the lower range of momentum transfer, but fails to predict the characteristic shoulder observed at higher values of *q*. Notice that the Gaussian model ignores the depletion of protein density at the core of the complex. In the second model, the protein is assumed to form a coat surrounding DNA with an inner and outer radius *r*_1_ and *r*_2_, respectively. The radial amino acid distribution is, hence, constant for *r*_1_ < *r* < *r*_2_ and given by $\rho (r) \, \pi \, (r_2^2-r_1^2) = 1$. The shell-like radial profile and corresponding model calculation are also shown in Figure 6. The cut-off in radial density at the outer radius of the coat results in a reasonable reproduction of the shoulder. In the fit procedure, the inner radius was set to the radius of the DNA molecule, that is *r*_1_ = 1 nm. The outer radius was optimized and takes the value *r*_2_ = 6.0 ± 0.5 nm. For both models, the contour distance between bound protein follows from the normalization and reads *L*/*N*_*p*_ = 10 ± 1 nm (i.e., 30 bps per Hfq hexamer).

## DISCUSSION

### Stretch of Hfq-incubated DNA

With increasing concentration of Hfq, the stretch of confined DNA along the direction of the channel decreases. This observation differs from what was previously reported for H-NS (42). In the case of H-NS, contraction is only observed at higher ionic strength (33 mM). At moderate ionic strength of 11 mM (T-buffer with 3 mM NaCl), the molecules elongate with increasing concentration of H-NS. The conformational response was interpreted in terms of polymerization of H-NS on DNA and H-NS mediated bridging interaction between different segments of the DNA molecule. Binding of H-NS results in an increase in persistence length to 130 nm for an H-NS dimer to bp ratio of 1:6. Bridging becomes effective only at higher ionic strength, related to increased screening of electrostatic repulsion between like-charged segments of the DNA molecule. In the case of Hfq, no elongation is observed. The absence of elongation is consistent with our atomic force microscopy results. The persistence and contour length were seen to increase and decrease, respectively, by 20% following binding of Hfq on DNA with a hexamer to bp ratio of 1:6. As shown by Monte Carlo computer simulation, these changes in contour and persistence length should result in a decrease of the stretch by ∼10% (42). Such a decrease in stretch agrees with the results obtained at moderate ionic strength irrespective channel cross-sectional diameter. However, at higher ionic strength, the observed decrease by 30–60% exceeds the prediction based on changes in persistence and contour length. Hfq mediated bridging becomes progressively more important and reduces the stretch at higher concentrations of salt. Bridging resulting in back folding, looping and side-by-side aggregation is also evident in the atomic force microscopy images of longer DNAs.

### Condensation of DNA by Hfq

For over-threshold concentrations of Hfq, the DNA molecules compact into a condensed form. Nanochannel facilitated condensation of DNA by neutral crowders, like-charge proteins (haemoglobin and bovine serum albumin) and H-NS has been reported before (38–39,42). The critical concentrations pertaining to Hfq and H-NS are at least two orders of magnitude lower than those for neutral crowders and like-charge proteins. Furthermore, there is a significant dependence on channel diameter, medium ionic strength and/or the presence of Mg cations. Hfq is much more effective in condensing DNA than H-NS. For instance, for DNA molecules inside 200 × 300 nm^2^ channels and in T-buffer with 3 mM NaCl, the critical concentrations are 0.005 μM Hfq and 0.5 μM H-NS. These values correspond with an Hfq hexamer and H-NS dimer to bp ratio of 1:1000 and 1:10, respectively. Hfq also condenses DNA in the bulk phase, but this requires an order of magnitude higher concentration. Unconstrained DNA cannot be compacted into a condensed form by H-NS in prevalent environmental conditions (42).

In view of the submicromolar concentrations of Hfq, the observed phenomena cannot be explained by macromolecular crowding. A plausible explanation for condensation is Hfq mediated bridging of segments of the DNA molecule, like multivalent polyamines bind and bridge DNA (58). For a transition to a condensed state, the segments should be (almost) parallel and juxtaposed. The confinement inside a nanochannel imposes orientation order, thereby increasing the attractive interaction resulting in the compaction into the condensed form. On the other hand, due to the linearization of the DNA molecule, the probability for segment juxtaposition is reduced. This reduction in segment juxtaposition results in a shift of the critical concentration for condensation toward higher values for smaller channel diameter. In the narrow channel and minimal screening conditions, the molecules do not condense for an Hfq-to-bp ratio less than 1:40. As in the case of contraction, condensation is facilitated by screening of the electrostatic repulsion between like-charged segments of the DNA molecule through an increase in ionic strength and/or binding of divalent magnesium.

### Structural arrangement of bound Hfq on DNA

The moderate increase in persistence length by 20% indicates that Hfq does not polymerize on DNA and no rigid filament is formed. Insight in the arrangement of bound Hfq can be obtained from a comparison of the known dimensions of the Hfq hexamer and the structural parameters derived from the scattering experiment. The hexameric core of Hfq can be modeled as a disk-like object with a diameter and a thickness of around 7 and 3 nm, respectively (13). The outer radius of the nucleoprotein complex of 6 nm indicates that the protein is not firmly docked in either the transverse or lateral direction. If the protein binds with the flat face of the core facing the duplex, the expected radial outer dimension would be <4 nm. In a transverse configuration, a larger outer radius exceeding 8 nm is expected. Our structural data do agree, however, with a dangling protein with some degree of freedom, possibly attached to the duplex through the C-terminal extension of the Hfq hexamer. Indeed, it has been suggested that the C-terminal tail could track along the grooves and interact with the phosphate backbone (5). Such a dangling protein would take a lateral distance along the contour of ∼10 nm and a radial distance away from the helical axis of the DNA molecule of ∼5 nm. The propensity for bridging can then be understood by binding of the protein, again possibly through one or more of the C-terminal extensions to other segment(s) of the DNA molecule. The 20% reduction in contour length following binding of Hfq might be related to opening of AT-rich tracks of the double helix (21).

## CONCLUSIONS

Hfq is limiting in the cell and RNA-related regulations compete with DNA binding. Nevertheless, RNA has been shown to be actively cycled *in vivo*, thus allowing effective regulation (59). Taking into account the lower affinity of Hfq for DNA compared to that of RNA and the high concentration of DNA inside the bacterial cell (bp concentration of ∼20 mM), it is most likely that Hfq is also actively cycled on DNA (21,60). Inside bacteria, the estimated concentration of Hfq bound to DNA is a few hundred nanomolar (26). This concentration falls within the range of concentrations in our experiments. We obtained similar results if the chloride salt is substituted by the more physiologically relevant potassium glutamate (61). Furthermore, the cross-sectional diameters of the nanochannels of a few hundred nanometer are comparable to the size of the bacterial nucleoid. Accordingly, we believe that our results have implications for the understanding of the function of Hfq in the regulation of the conformation and compaction of DNA *in vivo*.

Our experiments show that Hfq binds on double stranded DNA, but a rigid filament like in the case of binding of H-NS on DNA is not formed. Rather the nucleoprotein complex retains its flexibility with a moderate increase in bending persistence length. Hfq has a strong propensity for bridging of DNA segments, resulting in compaction and, eventually, a collapse to a condensed state for over-threshold concentrations of Hfq. Interaction between segments of the DNA molecule, controlled by electrostatic screening and segment orientation order imposed by nanoconfinement, plays a pivotal role. These observations indicate that Hfq is involved in the architectural organization of the genome, rather than regulation of gene expression *per se*. Our scattering data show that the protein is not firmly docked, but dangles at a radial distance of ∼5 nm from the helical axis of the duplex. Furthermore, we observed a decrease in contour length of the nucleoprotein complex with respect to the protein-free state. A plausible structural model is that the protein binds with the C-terminal extension to AT-rich sequences, which might require opening of the duplex with a concomitant decrease in contour length. Once bound, the dangling Hfq hexamer can bind one or more other DNA segments with the other C-terminal extensions and thus form a bridge with multi-arm functionality.

## SUPPLEMENTARY DATA

Supplementary Data are available at NAR Online.

SUPPLEMENTARY DATA
