# Supplementary material for: Effects of Hfq on the conformation and compaction of DNA
Source: Nucleic Acids Res. 2015 Mar 30;43(8):4332–41. doi: 10.1093/nar/gkv268 (PMC4417175; doi:10.1093/nar/gkv268)
Supplement: SUPPLEMENTARY DATA [file supp_43_8_4332__index.html]

Effects of Hfq on the conformation and compaction of DNA — SUPPLEMENTARY DATA 

# Effects of Hfq on the conformation and compaction of DNA

## SUPPLEMENTARY DATA

**Files in this Data Supplement:**

- SUPPLEMENTARY DATA
