# Supplementary material for: Effects of Hfq on the conformation and compaction of DNA
Source: Nucleic Acids Res. 2015 Mar 30;43(8):4332–41. doi: 10.1093/nar/gkv268 (PMC4417175; doi:10.1093/nar/gkv268)
Supplement: SUPPLEMENTARY DATA [file supp_gkv268_nar-00086-f-2015-File002.pdf]

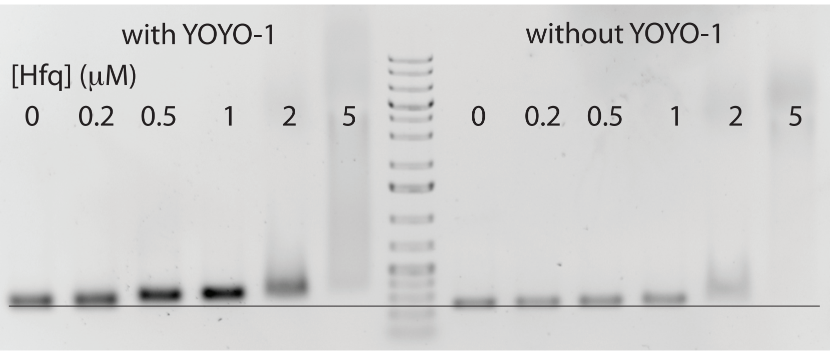

Figure S1: Electrophoretic mobility shift assay of 250 bp DNA incubated with YOYO-1 (50 bps per dye) and various concentrations of Hfq. The control is done without YOYO-1. Irrespective YOYO-1 and in agreement with equilibrium dissociation constant K_D measurements, the band shifts between 0.2 and 0.5 μM Hfq (21). Notice the brighter bands due to double staining and/or protein induced fluorescence enhancement (62). In particular, the latter effect confirms Hfq binding in the presence of YOYO-1

62. Hwang, H. and Myong, S. (2014) Protein induced fluorescence enhancement (PIFE) for probing protein-nucleic acid interactions. Chem.Soc. Rev., 43, 1221–1224.
